# Laptop computer induced erythema ab igne: a new presentation of an old disease*

**DOI:** 10.1590/abd1806-4841.20165139

**Published:** 2016

**Authors:** Ana Gabriela Salvio, Adauto Jose Nunes, Dora Patricia Ramirez Angarita

**Affiliations:** 1 Hospital Amaral Carvalho – Jaú (SP), Brazil; 2 Universidade Estadual Paulista "Júlio de Mesquita Filho" (Unesp) – Botucatu (SP), Brazil; 3 Universidade Federal de São Carlos (UFSCAR) – São Carlos (SP), Brazil

**Keywords:** Hot temperature, Hyperpigmentation, Infrared rays

## Abstract

Erythema ab igne is a condition characterized by skin changes due to chronic exposure to moderate temperature. We describe a female patient with continuous use of a laptop computer on exposed legs for 6 months and consequent development of reticulated hyperpigmentation at the area. Histopathological examination revealed epidermal atrophy, collagen fragmentation, and vacuolar changes in the basal layer, among other signs. We consider this case to be a modern cause of erythema ab igne.

## INTRODUCTION

Erythema *ab igne* is a condition characterized by reticulated macular hyperpigmented lesions caused by repeated exposure to moderate intensity heat. The condition has reemerged in the last years because of the increasing use of portable computers or laptops and other heat source apparatus. We report a case the erythema *ab igne* involving the use of a laptop computer.

## CASE REPORT

**Figure 1:**
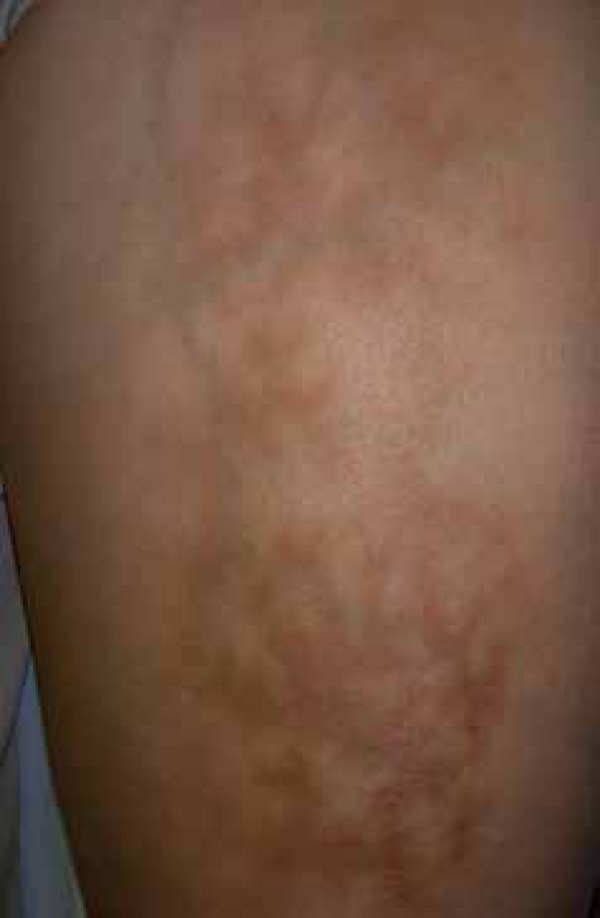
Reticulated, reddish- brown pigmented patch on the thigh of a female patient

**Figure 2:**
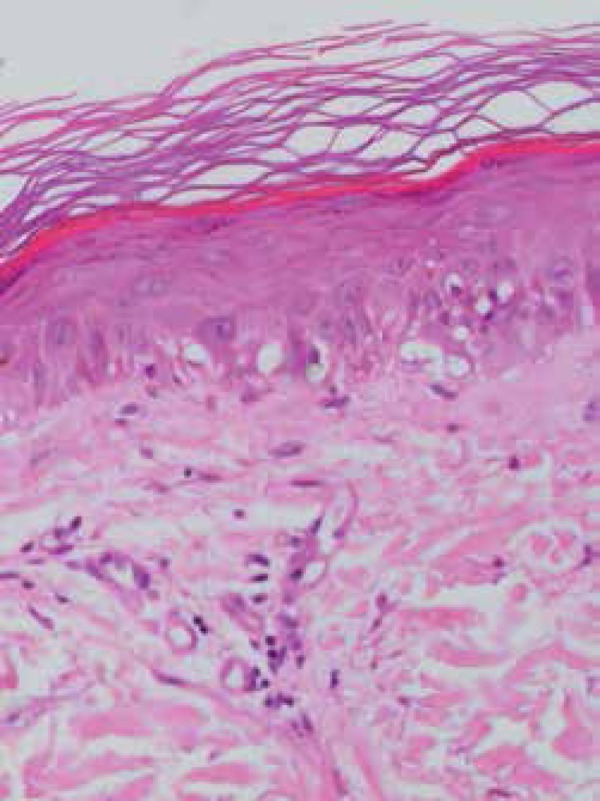
Epidermal atrophy with vacuolar degeneration of the basal layer, apoptotic keratinocytes and mononuclear perivascular infiltration (haematoxylin and eosin 100x)

A 55-year-old woman presented with a three-month history of an asymptomatic patch on both anterior thighs. Clinical examination revealed a well delimited area of reticulated hyperpigmentation (Figure 1). Patient history showed continuous use of a laptop computer on exposed thighs for 6 months. Epidermal atrophy, loss of dermoepidermal junction and vacuolar alterations in the basal layer are some of histopathologycal features of erythema *ab igne*. A collagen fragmentation associated to a perivascular infiltrate with melanin and hemosiderin deposition can also be present. Some cases exhibit hyperkeratosis and epidermal dysplasia.^1^ In the present case, we report epidermal atrophy, vacuolar degeneration of the basal layer, rare apoptotic keratinocytes and mild mononuclear perivascular infiltration (Figure 2).

## DISCUSSION

Erythema *ab igne* (EAI) is directly related to the chronic exposure of the skin to heat at a lower level than that which may cause a burn. An erythematous, reticulated, macular hyperpigmentation lesion develops at the site of exposure.^2^ Although it is usually asymptomatic, patients may describe local symptoms of burning or itching. A few cases described suggest that bullous EAI should be considered a well-defined variant of EAI.^3^ In the past, women who used firewood and coal stoves commonly presented this condition on the legs, the region directly exposed to the heat. Since the introduction of heating systems, the frequency of occurrence of this condition has decreased. Studies attribute EAI to the use of heat in the treatment of pain caused by chronic pathologies like Crohn's Illness, peptic pancreatic ulcer or metastasis.^4-5^ In modern society, the disease is related to new causative agents such as the use of sauna belts, slimming and anti-cellulite machines and, more frequently, the use of laptops.^6,7^ The capacity of laptops to generate heat depends on the model and central processing unit (CPU) components in the laptop.^8^ Most of the heat, which can vary from 80°C to 130°C, is generated by the central CPU, graphic processing unit (GPU), lithium battery, and CD/DVD inner motor.^8^ The high temperature, which is dissipated by a fan system, may produce skin changes like EAI. Total suspension of exposure to heat is the immediate and most important treatment. Tretinoin and hydroquinone have been used to reduce persistent hyperpigmentation. Malignant transformation into squamous cell carcinoma and Merkel cell carcinoma have been described.^9-10^. This case illustrates the association between EAI and exposure to heat the laptop.
